# Development of a novel motivational interviewing (MI) informed peer-support intervention to support mothers to breastfeed for longer

**DOI:** 10.1186/s12884-018-1725-1

**Published:** 2018-04-11

**Authors:** Rhiannon Phillips, Lauren Copeland, Aimee Grant, Julia Sanders, Nina Gobat, Sally Tedstone, Helen Stanton, Laura Merrett, Stephen Rollnick, Michael Robling, Amy Brown, Billie Hunter, Deborah Fitzsimmons, Sian Regan, Heather Trickey, Shantini Paranjothy

**Affiliations:** 1 Division of Population Medicine, Neuadd Meirionnydd, Cardiff University, Heath Park, Cardiff, CF14 4YS UK; 2 Centre for Trials Research, Cardiff University, Cardiff, UK; 3 School of Healthcare Sciences, Cardiff University, Cardiff, UK; 4 Royal United Hospitals Bath NHS Foundation Trust, Bath, UK; 5 Department of Public Health, Policy and Social Sciences, Swansea University, Swansea, UK; 6 Involving People Wales Public and Patient Involvement Representative, Cardiff, UK; 7 Centre for the Development and Evaluation of Complex Interventions for Public Health Improvement (DECIPHER), Cardiff University, Cardiff, UK

**Keywords:** Breastfeeding maintenance, Peer-support, Motivational interviewing, Intervention development, Complex intervention, Qualitative, Behaviour change wheel, COM-B

## Abstract

**Background:**

Many women in the UK stop breastfeeding before they would like to, and earlier than is recommended by the World Health Organization (WHO). Given the potential health benefits for mother and baby, new ways of supporting women to breastfeed for longer are required. The purpose of this study was to develop and characterise a novel Motivational Interviewing (MI) informed breastfeeding peer-support intervention.

**Methods:**

Qualitative interviews with health professionals and service providers (*n* = 14), and focus groups with mothers (*n* = 14), fathers (*n* = 3), and breastfeeding peer-supporters (*n* = 15) were carried out to understand experiences of breastfeeding peer-support and identify intervention options. Data were audio-recorded, transcribed, and analysed thematically. Consultation took place with a combined professional and lay Stakeholder Group (*n* = 23). The Behaviour Change Wheel (BCW) guided intervention development process used the findings of the qualitative research and stakeholder consultation, alongside evidence from existing literature, to identify: the target behaviour to be changed; sources of this behaviour based on the Capability, Opportunity and Motivation (COM-B) model; intervention functions that could alter this behaviour; and; mode of delivery for the intervention. Behaviour change techniques included in the intervention were categorised using the Behaviour Change Technique Taxonomy Version 1 (BCTTv1).

**Results:**

Building knowledge, skills, confidence, and providing social support were perceived to be key functions of breastfeeding peer-support interventions that aim to decrease early discontinuation of breastfeeding. These features of breastfeeding peer-support mapped onto the BCW education, training, modelling and environmental restructuring intervention functions. Behaviour change techniques (BCTTv1) included social support, problem solving, and goal setting. The intervention included important inter-personal relational features (e.g. trust, honesty, kindness), and the BCTTv1 needed adaptation to incorporate this.

**Conclusions:**

The MI-informed breastfeeding peer-support intervention developed using this systematic and user-informed approach has a clear theoretical basis and well-described behaviour change techniques. The process described could be useful in developing other complex interventions that incorporate peer-support and/or MI.

**Electronic supplementary material:**

The online version of this article (10.1186/s12884-018-1725-1) contains supplementary material, which is available to authorized users.

## Background

Extending the duration of breastfeeding remains a challenge, particularly in high-income countries and where there is a formula feeding culture [1–4]. Many women in the UK report that they stopped breastfeeding sooner than they would have liked to [5]. Women who are of white-British origin, those living in socio-economically deprived areas, and younger mothers are at higher risk of early breastfeeding discontinuation [5]. Given the health benefits of breastfeeding during the first two years of a child’s life (and beyond) for mothers and their infants [6, 7], new approaches are required to support mothers to breastfeed for longer.

Breastfeeding peer-support is an approach where support is provided to mothers by mothers who have personal experience of breastfeeding. Peers may be perceived to be more approachable than health professionals in some settings, as they have direct experience of the challenges of breastfeeding within a social context where it is not the norm, and can provide role-models that mothers can relate to [8, 9]. Breastfeeding peer-support can be an accessible way of providing more intensive support where it is needed most [10], but the theoretical basis, critical components, and optimal mode of delivery of breastfeeding peer-support interventions are poorly defined, resulting in considerable variation in how it is delivered [11]. Systematic reviews have shown that peer-support can improve breastfeeding initiation and maintenance in low and middle-income countries [2, 4, 12]. However, four UK based randomised controlled trials have not found breastfeeding peer-support to be effective in improving breastfeeding maintenance [13–16]. These UK studies tested low intensity breastfeeding peer-support interventions, whereas breastfeeding peer-support is more likely to be effective if it is intensive, delivered face-to-face, and starts early in the postnatal period, and it is unlikely to be effective if only offered to women who actively seek help [2, 17], as this may prevent the intervention from reaching the women who are most at risk of stopping breastfeeding.

A realist review [18] identified ten Randomised Controlled Trials [15, 19–27] and five quasi-experimental studies [28–32] of one-to-one breastfeeding peer-support interventions for breastfeeding continuation. Only two of the studies identified theoretical models underpinning their interventions, citing social support theory [27] and social cognitive theory [32]. Peer-supporters were described as role models, affirming and normalising experiences and empowering the mother to identify solutions that work for her [18]. Breastfeeding peer-support interventions generally aimed to address issues related to the mothers’ own capacity and resource (i.e. lack of knowledge, unhelpful beliefs and attitudes, low breastfeeding self-efficacy), and issues related to health professional support or capacity [18].

One approach that has been successfully used in peer-led interventions in other areas of healthcare is Motivational Interviewing (MI) [33]. MI is a counselling approach that aims to strengthen personal motivation for, and commitment to, a specific goal by eliciting and exploring the person’s own reasons for change within an atmosphere of acceptance and compassion [34]. The MI practitioner is trained to support the clients’ sense of autonomy, whilst evoking ‘change talk’ and softening ‘sustain talk’ [34]. In the case of breastfeeding maintenance, the desired behaviour is continuation of breastfeeding, while the behaviour to be changed is early discontinuation of breastfeeding, i.e. earlier than the World Health Organization (WHO) recommendations and/or before the mother’s own goals for breastfeeding continuation. MI is widely used in health care to help people resolve ambivalence about change, explore their concerns and set their own goals, and has proved effective in many areas of health care [34, 35]. There is evidence that MI can be used effectively in peer-led interventions, for example in supporting young people with HIV/AIDS, providing that adequate training and support are also provided [36, 37]. Using a MI based approach to breastfeeding peer-support could provide an opportunity to work in a person-centred and flexible way, which values mothers and fosters their autonomy, helping them to reach their breastfeeding goals. The application of the principles and techniques used in MI to the breastfeeding context (theory and practice) has not previously been systematically explored.

Developing a complex intervention that utilises both peer-support and MI-based techniques to support breastfeeding continuation requires an integrated framework to characterise the intervention and identify potential mechanisms. The Behaviour Change Wheel (BCW) [38] has increased in popularity in recent years and provides a unified and systematic framework for developing and characterising complex behaviour change interventions. The BCW framework outlines three stages of developing a behaviour change intervention: Stage 1 – understanding behaviour, Stage 2 – identifying intervention options, and Stage 3 – identifying behaviour change techniques and mode of delivery [39]. It can be used in a variety of ways to develop and/or characterise complex behaviour change interventions [39]. Within the BCW framework, the COM-B model helps to explain how interactions between people’s physical and psychological capability (**C**), social and physical opportunity (**O**), and automatic and reflective motivation (**M**) can influence behaviour [38]. The Behaviour Change Techniques Taxonomy V1 (BCTTv1) is used to identify and classify the content of behaviour change interventions [40]. The **a**ffordability, **p**racticability, **e**ffectiveness and cost-effectiveness, **a**cceptability, **s**ide-effects and safety, and **e**quity (APEASE criteria) are used within the BCW process as a control point to consider the feasibility of an intervention [39].

Breastfeeding peer-support may provide an accessible way of supporting mothers to breastfeed for longer, but breastfeeding peer-support is not well defined, its theoretical basis is unclear, and evidence of its effectiveness is mixed. The objective of this study was to develop a novel MI-informed breastfeeding peer-support intervention to support women to continue breastfeeding for longer in contexts where breastfeeding is not the social norm (i.e. younger women and those living in socio-economically deprived areas). We used the BCW framework to help us model and characterise the emerging user-informed intervention.

## Methods

**Fig. 1:**
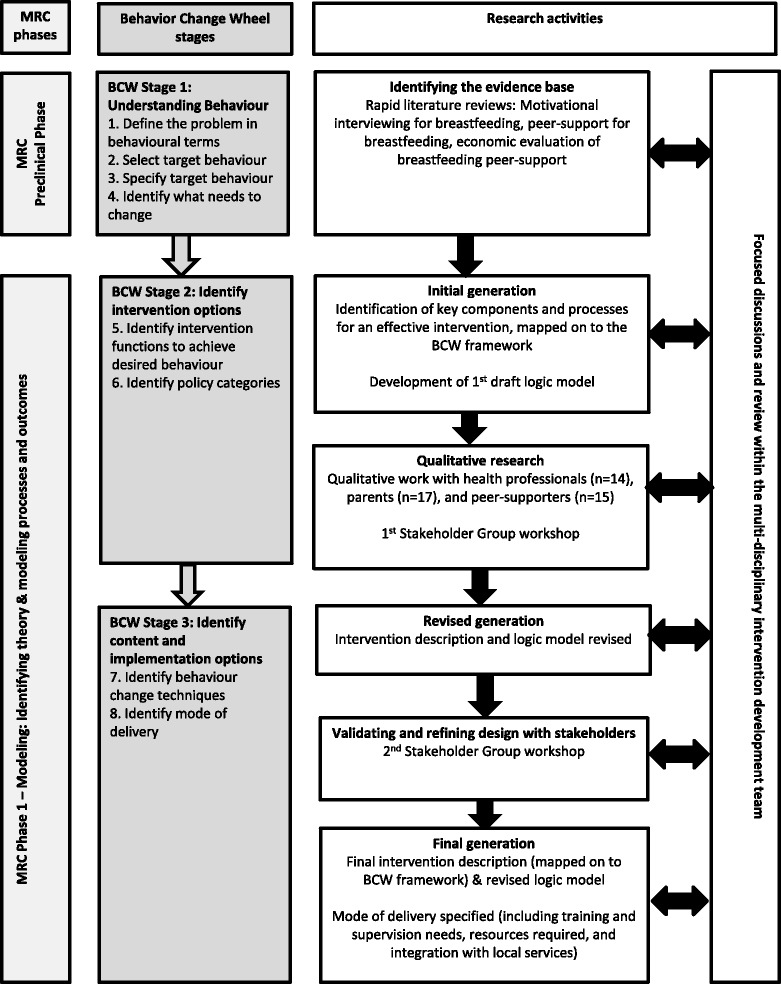
Overview of the MI-informed breastfeeding peer-support intervention development process

Following the BCW [39] and Medical Research Council (MRC) guidance on developing and evaluating interventions [41], we used a flexible and iterative intervention development process. This involved continuously re-visiting our theoretical understanding, logic model, and intervention design as we gathered and reviewed evidence from different sources. An overview of the intervention development process is provided in Fig. 1.

Our process for developing the intervention included use of published evidence [18], consultation with a stakeholder group, and qualitative work with potential service users and providers. Our multi-disciplinary and lay research team provided input throughout the process to integrate the breastfeeding peer-support and MI approaches, develop the content of training packages, and address potential implementation issues.

### Qualitative interviews and focus groups

The purpose of the focus groups and interviews was to inform the design of the intervention by helping us to understand the functions and acceptability of MI-based breastfeeding peer-support, and explore potential issues associated with implementation. We used focus groups conducted separately with mothers, fathers, and peer-supporters to identify experiences and perspectives within and across the groups, and identify areas of consensus and conflict [42, 43]. Semi-structured interviews were conducted with health professionals to obtain their views about their experiences of breastfeeding peer-support within their local service context, and to overcome practical difficulties in convening health professionals based in different geographical areas for focus groups.

#### Setting

Focus groups with parents took place in two sites in South Wales. Interviews with health professionals and focus groups with peer-supporters took place in the two South Wales sites, and at a site in the North West of England. These areas were selected for this project as they included communities with high levels of socio-economic deprivation, i.e. in the lowest quintile of area level social deprivation as measured by the Welsh Index of Multiple Deprivation [44] and the English Indices of Deprivation [45], and low breastfeeding rates (< 70% initiation). There were differing existing models of breastfeeding peer-support in the three areas. In South Wales, peer-support services were primarily voluntary and group-based. In the site in the North West of England, there was a more intensive and proactive one-to-one breastfeeding peer-support service in place, where peer-supporters were employed by the local children’s centre.

#### Participants and sampling

We conducted one focus group with fathers (*n* = 3) and two focus groups with mothers and pregnant women (*n* = 14). Mothers and fathers were recruited through existing community-based parenting groups in South Wales. Posters were distributed to parents via the group coordinators, inviting parents to take part in a focus group at a local venue at a specified date and time.

We carried out three focus groups with peer-supporters (*n* = 15) and a one-to-one interview with a peer-supporter, as only one individual had attended the planned group. An inclusion criterion was that participants had completed formal training in breastfeeding peer-support. An open invitation for participation in the focus groups was sent out to peer-supporters currently working in South Wales. The invitation was distributed via e-mail, telephone and social media, and was disseminated via informal peer-supporter networks, local midwifery service managers, breastfeeding peer-support coordinators, and using databases held by local health services of qualified breastfeeding peer-supporters. In the North West of England study site, the research team sent an e-mail invitation directly to the peer-supporters working within the local breastfeeding peer-support service.

We conducted 14 telephone interviews with health professionals whose role included breastfeeding support: health visitors (*n* = 2), midwifery service managers (*n* = 2), community midwives (*n* = 4), postnatal/hospital-based midwives (*n* = 3), an early years’ practitioner (*n* = 1), and midwifery support workers (*n* = 2). We recruited a stratified purposive sample [46] of health professionals involved in supporting breastfeeding in the participating areas, in different services (e.g. midwifery and health visiting), and at different levels of seniority within these services. Of the 18 purposively sampled health professionals who were invited to participate, 15 went on to take part in an interview.

#### Procedure

We developed flexible semi-structured topic guides, focusing on past experience of breastfeeding support, views on breastfeeding peer-support, views on the most appropriate timing and method of contact between mothers and peer-supporters, the training and support needs of peer-supporters, how partners should be involved by peer-supporters, what would encourage/discourage utilisation of a breastfeeding peer-support service, and how breastfeeding peer-support should be integrated with local services. The MI approach was briefly described to health professionals, who were asked for their views on this being used in breastfeeding peer-support. All interviews and focus groups were audio-recorded. All focus group participants provided written informed consent. Health professionals provided audio-recorded verbal consent for their interviews. Topic guides have are provided as Additional files 1, 2, 3 and 4.

#### Qualitative analysis

Qualitative data were transcribed verbatim, anonymised, and analysed thematically using an approach that was both deductive and inductive. An initial coding framework was developed using the BCW as a guide. This enabled us to map themes identified in the data against the different levels of the BCW (i.e. sources of behaviour, intervention functions, service/policy categories, and mode of delivery). Analysis was facilitated by the use of NVivo 10 qualitative software. The qualitative researchers (LC, HT, AG, RP) met regularly during the analysis process to discuss coding and interpretation of findings. A sample of transcripts (20%, one focus group and three interviews) was independently double-coded to assess the validity of the coding framework. Assessment of the dual-coded transcripts indicated a high level of agreement between coders. Where NVivo identified discrepancies of > 5% during dual coding, these codes were discussed and discrepancies resolved. Some themes in the initial coding framework were more explicitly defined, collapsed and re-labeled following this process to simplify the coding structure and ensure it fitted with the BCW definitions. Pseudonyms were allocated to participants to protect anonymity in reporting findings.

### Stakeholder consultation

A Stakeholder Group (*n* = 23) was convened to advise on all aspects of intervention development. This group consisted of: service users (*n* = 2), peer-supporters (*n* = 1), peer-support co-ordinators (*n* = 3), infant feeding co-ordinators (*n* = 1), service managers (*n* = 4), midwives (*n* = 1), health visitors (*n* = 2), MI trainers (*n* = 2), and voluntary sector representatives (*n* = 7). Two half-day creative workshops were held. In January 2015, the stakeholder group met to discuss preliminary findings from the literature review and qualitative research, and the initial framework for intervention that had been generated by the research team. Between January and March 2015, analysis of the qualitative work was used to inform the development of a more detailed specification of the intervention, which was presented to the Stakeholder Group in March 2015. The research team led the sessions and moderated group work. Group discussions were audio-recorded and key points extracted. Drafts of the intervention description and logic model were circulated to this group for comment between meetings. We also consulted with mothers who were waiting to be trained by the NHS as a peer-supporter and those going through the training using a closed Facebook group, including obtaining feedback on information for intervention recipients and the name of the intervention.

## Results

### Qualitative findings

#### Behaviour change wheel stage 1: Understanding behaviour

Using our qualitative findings, we compiled a list of potentially modifiable sources of behaviour for breastfeeding (dis) continuation, and mapped these against the elements of the COM-B model (Table 1). We verified and supplemented this with an informal review of the literature relating to factors associated with continuation of breastfeeding, which included motivation, self-efficacy, knowledge, skills, affective attitudes, social norms, social support, and beliefs that breastfeeding is a ‘normal’ and ‘healthy’ way to feed an infant [47–51].

**Table 1:** Sources of behaviour that could be targeted by breastfeeding peer-support and their corresponding COM-B domains

Sources of breastfeeding behaviour: barriers (−) and facilitators (+)	COM-B domain
Social norms: Bottlefeeding (−) or breastfeeding (+). Includes wider cultural/social norms, and beliefs and attitudes of significant others (e.g. partner, mother, sister) that bottlefeeding (−) or breastfeeding (+) is easier/convenient/healthier/more natural	Opportunity (social)
Feel comfortable (+) or uncomfortable (−) about breastfeeding in front of others/in public places	Opportunity (social & physical), motivation (automatic & reflective), and capability (psychological)
Social support: Social isolation (−) or feeling emotionally supported (+)	Opportunity (social)
Beliefs that bottlefeeding (−) or breastfeeding (+) is easier/ convenient/healthier/more natural. Beliefs/expectations about what is ‘normal’ breastfeeding (e.g. frequency of feeding, or how milk let down feels)	Capability (psychological)
Planning for bottlefeeding (−) or breastfeeding (+), e.g. buying equipment, formula, clothing	Opportunity (physical), motivation (reflective), capability (psychological)
Intention to breastfeed: determination to overcome challenges encountered (+) vs. intention to bottlefeed if there are difficulties (−)	Motivation (reflective), capability (psychological)
Confidence (+) and autonomy (+), e.g. feeling able to try out and find their own techniques for feeding rather than having to stick to ‘textbook’ methods	Motivation (reflective), capability (psychological)
Positive (+) or negative (−) prior experience of breastfeeding and/or breastfeeding support	Opportunity (physical), capability (psychological), motivation (automatic & reflective)
Quality of information and advice: Consistent (+) or inconsistent (−), and accurate (+) or inaccurate (−) advice and information from social and professional sources of support	Opportunity (social & physical), motivation (reflective), and capability (psychological)
Being able (+) or unable (−) to access support services at the right time (e.g. to plan/prepare prior to birth, soon after birth, at crisis points)	Opportunity (physical), capability (psychological & physical)
Physical factors, e.g. difficult birth (−), hospital environment (−), positioning (+/−), pain (−), latching (+/−), milk supply (+/−), frequent feeding (−), return to work or other separation from baby (−), managing siblings and other demands on time/resources (−), lack of sleep (−), change in routine (−), skin-to-skin contact (+).	Capability (physical & psychological)

#### Behaviour change wheel stage 2: Identifying intervention options

A summary of themes mapped against perceived functions of peer-support and views on implementation issues (mode of delivery) is provided in Table 2. Education, training, modelling, restructuring the social environment, and enablement were all perceived to be functions of breastfeeding peer-support interventions, although the emphasis on these different functions varied between the different stakeholder groups. The educational component of the intervention was a prominent theme across all groups. Consistency of information and advice provided to women was a recurrent theme for all participant groups:

**Table 2:** Summary of qualitative themes relating to functions and mode of delivery of breastfeeding peer-support

Intervention Functions	Mothers	Fathers	Peer-supporters	Health Professionals
Education *Increasing knowledge or understanding*	Consistency of advice is important but lacking. Want to be informed but not overloaded. Knowing what is ‘normal’ and what to expect is important.	Consistency of advice is important but lacking. Fathers wanted information themselves on breastfeeding and what they could do to support their partners.	Consistency of advice is important but lacking. Providing information and counteracting misinformation is an important part of the peer-supporter role.	Consistency of advice is important. Peer-supporters should be reinforcing and adding to advice provided by health professionals, not giving different information.
Training *Imparting skills*	Not a strong emphasis on this. Some mothers discussed being shown what to do after the baby had arrived. A few of the mothers said that they did not want to be physically touched when breastfeeding techniques were being demonstrated.	Felt that understanding more about breastfeeding techniques, e.g. by having a chance to try positions themselves during training using dolls, could help them to support their partners.	Giving mothers practical advice to help them develop their breastfeeding skills, particularly during the early post-natal period, was seen to be an important aspect of the peer-supporter role.	There was an emphasis on providing support to mothers with the technical aspects of breastfeeding, such as positioning and latch.
Modelling *Providing an example*	Peer-supporters, as mothers who have breastfed, can provide a more ‘realistic’ view of what to expect, what is ‘normal’ breastfeeding, and provide more than ‘textbook’ advice.	Being able to talk to somebody who had ‘been through it before’ and could share their experiences was considered useful.	Felt that sharing their own experiences was important in supporting mothers.	Thought it would be useful for mothers to be able to talk to somebody they can relate to, and who has recent experience of breastfeeding.
Restructuring the environment *Changing the social or physical context*	Providing social support is an important part of the peer-supporter’s role. Breastfeeding can be ‘isolating’. Having somebody you can relate to, who is ‘on your level’, and who is positive, encouraging and non-judgmental can be helpful.	Fathers had an important role in providing social and emotional support to their breastfeeding partners. Fathers felt that a more ‘friendly’ approach from peer-supporters could be helpful for their partners.	Providing social support was a prominent theme. Peer-supporters felt that belonging to a ‘breastfeeding community’ was important for mothers. Providing practical social support (e.g. accompanying to groups, facilitating access to services) was considered important.	Social support was not such a prominent theme in this group. A service manager noted that providing social support is important in deprived areas where breastfeeding is not the social norm and breastfeeding mothers may become ‘isolated’. A community midwife and a health visitor felt that peer-supporters could provide emotional support to mothers.
Enablement *Increasing means/reducing barriers*	Enabling access to other sources of support (e.g. engaging with and activating partners & introducing or accompanying mothers to groups) was an important part of the peer-supporter’s role.	Fathers wanted to play an active role in supporting mothers, and wanted to be included by health professionals and peer-supporters to enable them to do this. They wanted to have the knowledge and confidence to be able to seek help when it was needed.	Enabling mothers to access to other sources of support (e.g. engaging with and activating partners, introducing or accompanying mothers to groups, acting as an advocate) is perceived to be an important part of their role.	Peer-supporters were viewed as having an important role in acting as an advocate for the mother, for example in activating her social support network and in challenging negative attitudes of others towards breastfeeding.
Persuasion, incentivisation, coercion and restriction	Did not want to feel pressurised into breastfeeding. Persuasion can lead to feelings of ‘failure’. Style of communication is important, and should be positive and build autonomy and confidence.	Preferred collaborative to authoritarian approaches, expressing a desire for peer-supporters to be ‘supportive’ and ‘non-judgmental’. They felt that information should be balanced, neutral, and support their and their partners’ choices.	Peer-supporters did not think that pressurising or persuading mothers was acceptable or useful, and could result in mothers disengaging with breastfeeding support.	A few of the health-professionals stresses that peer-support should not be ‘judgmental’. One of the health professionals noted the importance of working with mothers in a way that did not make them feel guilty or as though they had failed if they ran in to difficulties.
Mode of Delivery				
Timing & frequency of contact	No set frequency or timing; flexible to meet mothers’ needs. Antenatal contact was viewed as being useful in getting information and building a rapport with the peer-supporter. Post-natal support should be provided early on, including on the post-natal ward. Mothers felt that the duration of the intervention should also be flexible, as mothers may need help further down the line with issues like weaning and returning to work.	Fathers felt that they would prefer to be given support after the baby was born than before, but they thought it might benefit their partners to have an opportunity to meet and develop a relationship with their peer-supporter before the baby was born. Fathers felt that support should be provided as soon as possible after birth until the bay is no longer being breastfed.	No set frequency or timing; flexible to meet mothers’ needs. Antenatal contact was viewed as being useful in providing information and building a rapport with mothers. Post-natal support should be provided early on, but access to hospitals could be difficult. Peer-supporters did not have a definite idea on when the intervention should end, but felt that mothers should be able to contact them for further advice or join local groups to provide longer-term support when breastfeeding is established.	No set timing or frequency; flexible to meet mothers’ needs. Support in the antenatal period was seen as important in developing a relationship and providing continuity of care. Early post-natal support was viewed positively by most (including in the hospital), although one post-natal midwife felt that it might be problematic to have another person providing support during this busy period.
Resources	Peer-supporters were viewed as having more time to spend with mothers than health professionals. It was recognised that boundaries around the peer-supporter role were important in ensuring they weren’t compensating for gaps in health care provision/support from mothers’ own social networks.	Peer-supporters were viewed as having more time to spend with mothers than did health professionals.	Most of the peer-supporters were currently working on a voluntary basis, but felt that to deliver a more intensive one-to-one service, being paid would make the job more viable (e.g. to cover childcare costs, or where their family relied on a second income). This would enable them to provide greater continuity of care and build up relationships with mothers.	Peer-support was viewed as something that should be provided in addition to, not in place of, existing services. Paying peer-supporters was viewed positively in terms of encouraging professionalism, but there were concerns about recent budget cuts, and having to divert resources away from other areas to fund it. Peer-supporters were seen as having more time and flexibility when working with mothers. There was a perceived demand for peer-support roles, but retention of peer-supporters and providing on-going training could be challenging given the pressures on maternity services.
Boundaries	One of the mothers acknowledged that peer-supporters might end up doing things that are outside of their role to compensate for gaps in care.	Not discussed.	Boundaries around working hours and availability of peer-supporters were felt to be important, as well as to what extent they should provide practical support (e.g. looking after a baby for a mother to have a shower when she is feeling desperate).	Boundaries were felt to be important, particularly in relation to availability of peer-supporters and working hours. It was felt that this was more pertinent in a one-to-one service as opposed to a group support setting.
Training and support	Peer-supporters should have had relevant police checks, adequate training, and be connected with a wider team of health professionals.	Felt that somebody with personal experience of breastfeeding was important, but did not specify any other training requirements for peer-supporters.	Peer-supporters felt that good training, supervision and relationships with health care providers would be essential in delivering a one-to-one service to mothers. Having relevant police checks and appropriate training in safeguarding and local NHS policies and procedures (e.g. hand washing policies) was considered essential	Training in communication skills and listening skills, was viewed as important. An MI based approach was viewed as being useful for building mothers’ confidence, helping them understand barriers to breastfeeding, and ‘looking at the positives not the negatives’. Peer-supporters would need relevant police checks and training in safeguarding/ local policies and procedures. Health professionals felt that formal training that was in line with UNICEF Baby Friendly standards was required to ensure quality and consistency of advice.

*“But I think in the early days, before I joined this (parenting) group, I had like eight different people telling me how to feed. And they were all different. Whereas when you come to this group, you might get a different midwife, you might get a different health visitor, but they are saying the same sort of thing.”*

[Maya, mother]

Pregnant women and mothers tended to place a greater emphasis on modelling and social support, whereas fathers and health professionals focused more on training.

*“I think the social aspect is really important, I think it’s the main key, because as a breastfeeder you feel quite isolated, whether it’s within your family, within your friends, so belonging to a breastfeeding community gives you the encouragement to keep breastfeeding and to keep following what you want to do. I think without this community most of us probably wouldn’t have got anywhere as far on our own.”*

[Sally, peer-supporter]

For one of the service managers, peer-support was seen as being particularly valuable in counteracting negative attitudes in social contexts where breastfeeding is not the norm:

*“And quite often you’ll have mothers being told by relatives and friends, “Are you feeding again, he’s hungry again, you only just fed him”, you know, this type of thing, and of course the mothers get very demoralised then, and they start questioning the breastfeeding, they start thinking that you know, they’re not producing enough milk and all this sort of stuff. It takes quite a strong willed woman, and if she’s a young girl...”*

[Wendy, service manager]

There was a consensus across the groups that persuasion and coercion were not acceptable in the context of breastfeeding peer-support; pressurising mothers was contrary to the expressed needs of women and was viewed as being counter-productive. Mothers and fathers expressed a preference for a supportive, collaborative relationship, and valued positivity and encouragement. They expressed a preference for neutral and realistic information, rather than a persuasive approach that emphasised the benefits of breastfeeding.

*“You don't want that person preaching to you saying, oh, I breastfed my child until 18 months or whatever. You don't want that. You just want ‘I'm here to help”.*

[Alana, mother]

All participant groups viewed engaging with and activating mothers’ own support networks, both in terms of their social networks and other health care and community services that might be available to them, as an important element of peer-support. Peer-supporters acknowledged the need to engage with fathers in achieving this:

*“Maybe we need more support for the fathers then, so that they can provide that support to their partners in the early hours, you know when they can’t get to the peer supporter, call the fathers to have enough support and help them through it.”*

[Isla, peer-supporter]

From the parents’ perspective, another important aspect of the peer-supporters’ role was to link mothers with other services, including health professionals and local groups.

#### Mode of delivery of the breastfeeding peer-support intervention

Four core themes were identified in relation to implementation of the intervention (i.e. ‘mode of delivery’); the timing and frequency of contact between peer-supporters and mothers, resources, boundaries, and training and support for peer-supporters.

##### Timing and frequency of contact

In all participant groups, people felt that having initial contact between peer-supporters and mothers during the antenatal period would be beneficial, mainly in terms of building up a rapport. During the postnatal period, early support was often viewed as being important:

*“It only takes one little comment in an early stage to put the seed of doubt in somebody's mind and they think, ‘oh, I'm not doing this right.’ ‘I'm not making enough milk’ and that's the whole breastfeeding journey come to an end because of somebody's attitude early on, the throw away comment.”*

[Mia, peer-supporter]

Mothers, fathers and peer-supporters generally felt that peer-support should be able to continue in a flexible way until mothers had stopped breastfeeding. Peer-supporters felt that ending the intervention should be handled sensitively so that mothers didn’t ‘just feel dumped’.

##### Resources

Across all participant groups, there was an acknowledgement that maternity services were very busy and over-stretched, and there was a perception that peer-supporters would have more time to spend with mothers than health professionals, particularly during the critical early stages of breastfeeding. Many peer-supporters felt that post-natal support would ideally start while mothers were in hospital for this reason. Although the clinical context was not known, one of the mothers spoke about her experience of being on a busy post-natal ward in negative terms:

*“There was a lady opposite me absolutely sobbing her heart out because the midwife just wouldn't help her. You have to give her a bottle, you have to give her a bottle. She said, I don't want to give her a bottle, I want to feed her.”*

[Alana, mother]

Health professionals had more mixed views about providing breastfeeding peer-support in the hospital environment; some felt the hospital environment was too busy and pressured already and that ‘another body’ would not be helpful.

Employing peer-supporters, as opposed to a voluntary service, was generally seen as being a positive thing in making the work more viable for local women and providing more of a professional relationship between peer-supporters and local healthcare services. However, there were some concerns about diverting limited funding away from existing services.

##### Boundaries

Peer-supporters felt it was important for there to be boundaries around contact with mothers, particularly in terms of working hours and availability. This was a complex issue, with tension between wanting to provide flexible and responsive support, and needing to make the role practical and manageable. Mothers and health professionals also acknowledged the importance of setting boundaries around the peer-supporters’ role. Good communication between peer-supporters and health professionals, clear roles and responsibilities, good training and support, and familiarity with the local service context were viewed as being important in maintaining boundaries and facilitating integration with local services.

##### Training and support for peer-supporters

The health professionals, who felt that training needed to be robust, discussed training for peer-supporters most extensively. When we asked health professionals what they thought of using an MI-informed approach in breastfeeding peer-support, they were generally familiar with the basic principles of MI and felt that it would be useful, particularly in terms of developing communication and listening skills. They felt that MI would reinforce mothers’ confidence, help with ‘*looking at the positives not the negatives*’, and could ‘*draw out people's personal reasons and what they might be worried about’*. A health visitor said that *‘I get the impression it’s a very gentle approach, going with the flow type of thing’.*

### Stakeholder group consultation

Key themes from a realist review carried out by our team [18] and other relevant reviews [2, 17, 52], and the qualitative findings of this study were discussed with the Stakeholder Group. Key learning from these discussions was that:

1. Peer-supporters should: make **at least one antenatal contact** with pregnant women to enable information exchange and build rapport, make **contact with** **mothers in the first few days** after birth (to include around 72 h when babies are routinely weighed), provide **flexible on-going support** in the postnatal period to meet individual mothers’ needs, and the peer-supporters should **end the intervention in a way that provides affirmation** of the mother’s efforts and enables her to access other sources of support in the longer term (e.g. breastfeeding groups, online communities).
2. Boundaries around the peer-supporters’ role should be: clearly set from the outset; generated by an external party to provide consistency and ensure safety; acknowledge the limits of peer-supporters’ knowledge and skills, and; be discussed and reflected on during supervision sessions.
3. The intervention should focus on enabling mothers to cope outside of the peer-supporters’ working hours by signposting to other services and activating their social networks. For example, by including partners or other family members in discussions if they are present when peer-supporters visit mothers.
4. Responsibility for peer-supporter training needs to be clear, and appropriately resourced. Health professionals felt that training of peer-supporters was usually a community based activity and typically fell within the remit of health visitors.
5. Following initial training, peer-supporters need support from midwives and health visitors to help with their practice and to deal with any issues or questions that they are uncertain about. Peer-supporters should be linked up with each other, e.g. by having a meeting once a month where they can share experiences and good practice and through social media groups, such as closed Facebook groups.

The Stakeholder Group developed ‘principles of good practice’ for the delivery of MI based breastfeeding peer-support, stating that peer-supporters should be ‘supportive’, ‘positive’, ‘non-judgemental’, ‘approachable’, ‘honest’, ‘down to earth’, and ‘a good listener’. The intervention name ‘Mam-Kind’ was developed in conjunction with the stakeholder group and the peer-supporters who were part of the informal closed Facebook group set up for this study. The features of the intervention name that were important to stakeholders were that was centred on the mother, emphasised kindness, and did not include direct reference to breasts, breastfeeding, or breastmilk which were perceived to be off-putting for women in cultural contexts where breastfeeding was not the social norm.

### Final specification of the ‘Mam-kind’ intervention

**Fig. 2:**
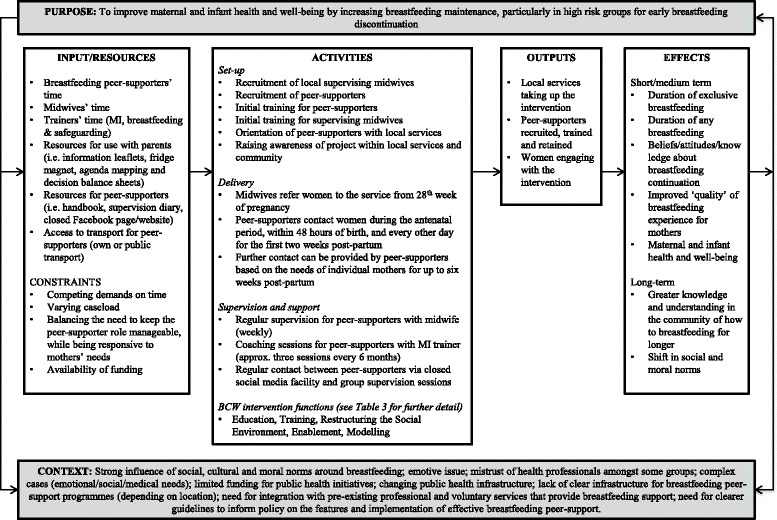
MI informed breastfeeding peer-support logic model

We developed the logic model for the intervention based on the results from our qualitative research and discussions with the Stakeholder Group (Fig. 2).

We produced a description of the content and timing of intervention sessions during the antenatal and postnatal periods and classified the behaviour change techniques included using the BCTTv1 (Table 3).

**Table 3:** Content of MI-base breastfeeding peer-support during the antenatal and postnatal periods and corresponding BCTTv1 techniques

Mode of delivery	Scope of session content	Intervention functions	Behaviour change techniques (BCTT v1)
Antenatal period	Engagement and building a rapport with mother and significant others (if present)	Restructuring social environment	Social support (unspecified)
Face-to-face visit (or telephone if this is a mother’s preferred option)			
	Information about accessing the intervention: what it’s about, how it works, letting us know when their baby has arrived	Education, training	Instruction on how to perform a behaviour
	Discuss an agenda with mothers: what can they expect and what they would like to get from the program	Enablement	Action planning
	Affirmation of the mothers’ strengths and capability, emphasising her autonomy	Enablement, restructuring social environment	Social support (emotional), social reward
	Explore mothers’ current knowledge and information needs and provide information as appropriate. Use open questions, reflective listening, and elicit-provide-elicit approaches to exchanging information with mothers	Education, training	Instruction on how to perform a behaviour, information about health, and social, environmental and emotional consequences
	Guide mothers in understanding their beliefs, motivations and intentions with regard to breastfeeding. Strengthen ‘change talk’ about breastfeeding and soften sustain talk about not breastfeeding	Education, Enablement	Identity associated with changed behaviour, framing/reframing, incompatible beliefs, pros and cons, goal setting (behaviour and outcomes), self-talk
	Planning for breastfeeding (e.g. how to overcome difficulties, how to get support)	Training, enablement	Instruction on how to perform a behaviour, problem solving, action planning
Postnatal period	Engagement & building a rapport – introductions, congratulations on the new arrival (first visit), seek collaboration. Convey empathy, affirm mothers’ strengths and capability, and emphasise her autonomy	Enablement, restructuring social environment	Social support (unspecified and emotional), social comparison, social reward, demonstration of behaviour
Face-to-face visit within 48 h of birth, either in hospital or at home (or contact by phone/text if this is not possible) Contact at least every other day (face-to-face, by phone, or by text) from days 3 to 14, including a visit close to the 72 h weighing of the baby			
	Use open questions and reflective listening to elicit from the mother how she is doing, how the feeding is going, and what support (if any) she would like. Explore ambivalence and concerns, and identify potential barriers and facilitators to continued breastfeeding. Provide information and skills training based on individual needs on breastfeeding relevant to the first few days and weeks	Education, training, enablement	Review behaviour & outcome goals, instruction on how to perform a behaviour, information about health, and social, environmental, and emotional consequences, identity associated with changed behaviour, pros and cons, framing/reframing, incompatible beliefs, social support (practical)
	Provide a role model for continued breastfeeding	Modelling	Demonstration of the behaviour
	Normalising experiences	Restructuring social environment	Social comparison
	Strengthen ‘change talk’ about continuing to breastfeed and soften ‘sustain talk’ about discontinuing breastfeeding earlier than the mother would like to	Enablement	Commitment, self-talk
	Planning for overcoming barriers to breastfeeding	Enablement	Problem solving, action planning
Ending the intervention	Use open questions and reflection to elicit from mothers what other sources of breastfeeding support they might need now and in the longer term. Signpost/refer to relevant services, act as an advocate when required. Offer practical support to overcome barriers to accessing support, such as accompanying mothers to a breastfeeding group or to a public place (e.g. local café) if they have concerns about feeding in public	Enablement, training	Action planning, instruction on how to perform a behaviour, social support (practical)
Provide a graded exit from the intensive one-to-one service from 2 weeks onwards			

The intervention was designed to allow sufficient flexibility to meet individual mothers’ needs, for it to be practical to deliver it, and for the behaviour change techniques included to be clearly defined and categorised. Having characterised the core functions and content of the intervention, we developed training materials for use with peer-supporters to provide them with the skills and knowledge required (available from authors on request).

## Discussion

We used a systematic and user-informed approach, guided by the Behaviour Change Wheel (BCW), to develop and characterise a novel MI-based peer-support intervention to support breastfeeding maintenance: the Mam-Kind intervention. The intervention developed in this study has important features for effectiveness and successful implementation that have been identified in previous literature, including being pro-active, intensive, mother-centred, and having a clear theoretical basis [2, 17, 18]. There is considerable variability in the provision of breastfeeding peer-support in the UK [11], and attention needs to be given to providing consistent, equitable, and evidence-based services that can reach those who are most at risk of early breastfeeding discontinuation. Implementation of the intervention, including supervision arrangements and training for supervising midwives, needs to be planned on a local level to allow the intervention to be integrated with local services that may vary in their structure, resources available, and level of need.

The BCW is an extensive but not exhaustive model [39]. MI is a complex intervention and there are several techniques used within MI that can be mapped on to behaviour change techniques included in other taxonomies [53]. However, much of MI focuses on relational and interpersonal techniques, known as ‘the MI spirit’, which can strongly influence its effectiveness [34, 54]. The Behaviour Change Technique Taxonomy version 1 (BCTTv1) focuses on technical behaviour change methods, such as goal setting and problem solving, and pays less attention to interpersonal-relational issue. Therefore, as previously noted when considering this system in the context of MI [53], we found that some adaptation of some BCTTv1 categories was required to fit with the MI approach.

The ethos of MI focuses on working with people, rather than doing things to them, to elicit change. For example, categories of the BCTTv1 related to the provision of information, but not to eliciting information from people thus drawing on their own knowledge and experiences. Using a MI informed approach, a peer-supporter would seek to guide a mother in reflecting on her breastfeeding goals and outcomes, using open questions and simple and complex reflections to explore her own beliefs, motivations, and ambivalence, which broadly fits within the ‘review behavioural goals’ and ‘review behavioural outcomes’ BCTT v1 categories. ‘Commitment’ is described in the BCTTv1 as asking a person to explicitly affirm or reaffirm their commitment to behaviour change. In a MI informed intervention, the approach is less directive, and this is affirmation of commitment is likely to come from the mother herself. We categorised affirmation as ‘social reward’ using the BCTTv1, as it provides positive reinforcement of the mother’s efforts. We categorised conveying empathy and emphasising autonomy as ‘social support (emotional)’ as these were not explicitly described in the BCTTv1.

We worked with the Stakeholder Group to develop a set of ‘guiding principles’ for our intervention to be used alongside the technical behaviour change techniques categorised using the BCTTv1, which allowed us to describe factors relating to its underlying ethos and inter-personal style. Our study has provided stakeholder support for a guiding principle that breastfeeding peer-support should focus on empowering mothers, supporting them through the breastfeeding journey, affirming their efforts and respecting their choices. This study indicated that very directive approaches to increasing breastfeeding maintenance and those that reduce the autonomy/control are likely to have low acceptability to mothers. These important, subjectively experienced factors apply to many types of intervention and can be challenging to objectively measure and assess, but need to be given sufficient attention in future iterations of the BCTTv1.

### Limitations

Limitations of the study are that a small number of local sites were used for the in-depth qualitative and stakeholder work (two sites in Wales and one in England). The MI-informed breastfeeding peer-support intervention that we have developed is intensive and would require more resources to deliver than low intensity/group based peer-support. Providing the feasibility and acceptability of the intervention can be demonstrated, evaluating the cost-effectiveness, as well as clinical effectiveness, of the intervention will be essential in future studies.

## Conclusions

Using a systematic and user-informed process, guided by the BCW, we have developed and characterised a novel MI informed peer-support intervention that can be tested for feasibility of delivery. The intervention development process described is likely to be useful in developing other interventions that use peer-support and/or MI-informed approaches, but the BCTTv1 system requires some adaptation to incorporate important inter-personal relational aspects of such interventions.

## Additional files

Additional file 1: Interview topic guide: development focus groups with mums (ante-natal and post-natal). (DOCX 26 kb)

Additional file 2: Interview topic guide: Development focus groups with dads (ante-natal and post-natal). (DOCX 25 kb)

Additional file 3: Interview topic guide: health professionals and service managers. (DOCX 477 kb)

Additional file 4: Interview topic guide: focus groups with peer supporters. (DOCX 26 kb)

## Abbreviations

BCTTv1: Behaviour Change Technique Taxonomy version 1
BCW: Behaviour Change Wheel
COM-B: Capability, Opportunity and Motivation model of Behaviour
MI: Motivational Interviewing
