# Supplementary material for: Development of a novel motivational interviewing (MI) informed peer-support intervention to support mothers to breastfeed for longer
Source: BMC Pregnancy Childbirth. 2018 Apr 11;18:90. doi: 10.1186/s12884-018-1725-1 (PMC5896150; doi:10.1186/s12884-018-1725-1)
Supplement: Supplementary file 1 — Interview topic guide: development focus groups with mums (ante-natal and post-natal). (DOCX 26 kb) [file 12884_2018_1725_MOESM1_ESM.docx]

**Motivational Interviewing based peer support for breastfeeding**

**Interview topic guide: development focus groups with mums (ante-natal and post-natal)**

**Introduction**

Thanks very much for coming to talk to us today.

We’re working on developing a new programme to support mums who are breastfeeding their babies.

To help us design this new programme, we’d like to find out what mums and mums-to-be think.

**[**Interviewer will ask the group to introduce themselves, and to tell the group about children that they have or are expecting.]

**Views on current provision of breastfeeding support**

1. Could you tell us a bit about how you have fed, or are thinking about feeding, your baby?

[Prompts: what are your thoughts about bottle or breastfeeding? What was your experience like? How did it make you feel? What support have you had around your feeding decision and following through on that decision?]

**Views on the proposed peer support programme**

In the programme we’re developing, mums who have chosen to breastfeed would be supported by other mums who have been given training in how to help mums breastfeed (peer-supporters).

1. What do you think about mums receiving support from other mums with breastfeeding (sometimes called breastfeeding peer-support)?

[Prompts: What would you want this support to be like? How should peer-supporters help mums? When do mums need this sort of help? How much contact should they have? Should this support be one-to-one or in groups? Face-to-face or by phone/text, or other methods of contact]

1. We are planning on giving our peer-supporters training in counselling. This would help peer-supporters to have supportive conversations with mums about breastfeeding. The conversations would focus on what mums want to do when it comes to breastfeeding, and on coming up with ideas to help her to achieve her goals. What do you think about this?

[Prompts: Would it be useful? When do you think it would be useful for mums to be able to talk things through with another mum? Are there key points where this sort of support would be useful?]

1. Mums who can get this new peer-support service would be told about the project by their midwife at 28 weeks pregnant. If they were interested, they would be asked for permission to pass their phone number on to the peer supporter. How does this sound to you?

[Prompt: Can you think of any other ways of recruiting pregnant women? Are there problems with recruiting mums in this way? From your perspective, what are the pros and cons of this approach?]

1. If you were going to take part in this program, when would you want to first meet with your peer-supporter?

[Prompts: Would this be before or after the baby is born?]

1. At the moment, we’re thinking about getting peer-supporters to contact mums who want to use the service a few weeks before the baby is born (at about 37 weeks pregnant). What do you think about this?
2. Imagine you are 37 weeks pregnant and you are meeting your peer supporter for the first time, what would you like to talk about?

[Prompts: What would you like your peer supporter to talk about? What would you like the outcome of this conversation to be? Would you like to be given information and if so what information would you like? How would you like the peer supporter to discuss this information with you?]

1. We are planning to have a peer-supporter visit mums within the first two days of baby being born. How do you feel about this?

[Prompts: Would it be OK for a peer-supporter to contact mums in the first couple of days after birth? Is a home visit ok? Is contact by phone or text OK?]

1. Peer-supporters would also visit every few days for two weeks after the baby is born, as well as keeping in touch by phone or text. What do you think about this?

[Prompts: How much contact should they have? What is the best way to contact mums?]

1. After the first couple of weeks, the peer-supporter wouldn’t get in touch with mums, but mums would still be able to contact their peer-supporter if they wanted to. What do you think about this?

[Prompts: Is it OK to leave it up to the mums to make contact after the first couple of weeks? How long do mums need the more intensive support for?]

1. We know that in the early days, just after the child is born, mums can have mixed feelings about their feeding choice. This can be very normal. Imagine that you had decided to breastfeed your baby and were then having second thoughts. So part of you really wanted to keep going and another part of you was just struggling with it. What kind of help would you like from a peer supporter?

[Prompt: What would you like her to do? How would you like her to talk with you? What would you like her not to do? What would really annoy or upset you? Would you like to receive any information and if so what would you like to know?]

1. We know that some mums may make the decision to stop breastfeeding in the weeks after giving birth. Imagine you have made the decision to stop breastfeeding. What would you like from your peer supporter?

[Prompt: what would you like her to do? How would you like her to talk with you? What would you like her not to do? What would you like the outcome of this conversation to be? What would really annoy or upset you? Would you like to receive any information and if so what would you like to know?]

1. Do you have any ideas about the sort of name that the peer support programme should have?

[Prompts: something breastfeeding related (bosom buddies, milk mates); something non-specific (little stars, acorns), other type of name]

1. If you were offered this type of peer-support service for breastfeeding, would you use it?

[Prompts: Why? Why not?]

1. What would put mums off having peer-support for breastfeeding?
2. What would encourage mums to use peer-support for breastfeeding?
3. If you were taking part in a breastfeeding peer-support programme, would you want your partner to be involved?

[Prompts: How? Why? Should/could partners or family members be there when peer-supporters are talking to mums? What information would partners or family members need and when? What is the best way to get partners or family members interested?]

**Closing questions**

1. Do you have any other comments?
2. Would you like a copy of the findings of our research?

Thank you very much for taking part.
