# Supplementary material for: Development of a novel motivational interviewing (MI) informed peer-support intervention to support mothers to breastfeed for longer
Source: BMC Pregnancy Childbirth. 2018 Apr 11;18:90. doi: 10.1186/s12884-018-1725-1 (PMC5896150; doi:10.1186/s12884-018-1725-1)
Supplement: Supplementary file 2 — Interview topic guide: Development focus groups with dads (ante-natal and post-natal). (DOCX 25 kb) [file 12884_2018_1725_MOESM2_ESM.docx]

**Motivational Interviewing based peer support for breastfeeding**

**Interview topic guide: Development focus groups with dads (ante-natal and post-natal)**

**Introduction**

Thanks very much for coming to talk to us today.

We’re working on developing a new programme to support mums who are breastfeeding their babies.

To help us design this new programme, we’d like to find out what Dads think.

**[**Interviewer will ask the group to introduce themselves, and to tell the group about children that they have or are expecting.]

**General views about breastfeeding**

1. What do you think about breastfeeding in general?

[What springs to mind when people talk about breastfeeding? What are the good and bad things about breastfeeding a baby? Do you have any concerns or questions about breastfeeding?]

**Views on breastfeeding support**

1. Were your children breastfed or bottlefed?

(For Dads from antenatal groups: If your partner is expecting a baby, is she thinking about breastfeeding or bottlefeeding?)

[Prompts: Did your partner have any difficulties with breastfeeding?]

1. Has your partner had any help with breastfeeding?

(If antenatal, this includes information or advice about breastfeeding]

[Prompts: What about health professionals; peer-supporters; other mums; family? What did you think about the support she received? What were the best and worst things about the support? What additional support would you/your partner have liked?]

1. Does the support that’s given at the moment include Dads?

[Prompts: Has anyone spoken to you about breastfeeding? Have you been with your partner when someone has talked to her about breastfeeding (e.g. a midwife, health visitor, or another mum). Have you been to any groups or clinics for breastfeeding with your partner?]

**Views on the proposed new peer-support programme**

In the programme we’re developing, mums who breastfeed would be supported by other mums (called peer-supporters) who have been given training in helping mums with breastfeeding.

1. What do you think about mums getting support from other mums with breastfeeding?

[Prompts: What sort of support could mums get from other mums? How is this different from getting help from health professionals, like midwives? When would be the best time for mums to get support from other mums with breastfeeding?]

1. In this new programme, a peer-supporter would get in touch with mums a few weeks before they have their baby. What do you think about mums meeting their peer-supporter before the baby is born?

[Prompts: Is this a good time for mums to start getting information on breastfeeding? Do Dads need information on breastfeeding before the baby arrives?]

1. Imagine your partner is 37 weeks pregnant and she is meeting the peer supporter for the first time, what do you think she might like to talk about to the peer supporter?

[Prompts: What do you think she might like the peer supporter to talk to her about? What do you think she would like as the outcome of that conversation? Do you think she would like to receive information and if so what information do you think she would like? How do you think she would like the peer support to discuss this information with her?]

1. Mums would get quite a lot of support in the first couple of weeks after the baby is born. The peer-supporter would visit mums within the first two days of baby being born. What do you think about this?

[Prompts: Would this be OK for mums? What about Dads? Should Dads be around when the peer-supporter visits]

1. The peer supporter would also visit every few days in the first couple of weeks, as well as keeping in touch by phone or text if needed. What do you think about this?

[Prompts: Would this be OK for mums? What about Dads? Should Dads be around when the peer-supporter visits]

1. After the first couple of weeks, the peer-supporter wouldn’t get in touch with mums, but mums would still be able to contact their peer-supporter if they wanted to. What do you think about this?

[Prompts: Is it OK to leave it up to the mums to make contact after the first couple of weeks? How long do mums need the more intensive support for?]

1. We know that in the early days, just after the child is born, mums can have mixed feelings about their feeding choice. This can be very normal. Imagine that your partner has decided to breastfeed your baby and was then having second thoughts. So part of her really wanted to keep going and another part of her was just struggling with it. What kind of help do you think your partner would like from a peer supporter?

[Prompt: What do you think she would like her to do? How do you think she would like the peer supporter to talk to her? What do you think she would like the peer supporter not to do? What do you think the peer supporter could do to really annoy your partner? Do you think your partner would like to receive any information and if so what would she like to know?]

1. We know that some mums may make the decision to stop breastfeeding in the weeks after giving birth. Imagine your partner has made the decision to stop breastfeeding. What do you think she would like from her peer supporter?

[Prompt: what do you think your partner would like the peer support to do? How do you think your partner would like the peer support to talk to her? What do you think she would like as the outcome of that conversation? What do you think she would like the peer supporter not to do? What do you think the peer support could do that would really annoy or upset her?]

1. Should Dads be included in peer-support?

[Prompts: How? What is Dads’ role in supporting mums who are breastfeeding? What support do Dads need?]

1. How could we get Dads involved in a programme that supports breastfeeding mums?

[Prompts: What is the best way to get Dads interested? What’s the best way to involve them? What would encourage you to take part? What would put you off? Should/could Dads be there when peer-supporters are talking to mums?]

1. Do you have any ideas about the sort of name that the peer support programme should have?

[Prompts: something breastfeeding related (bosom buddies, milk mates); something non-specific (little stars, acorns), other type of name]

1. Do you have any other comments?
2. Would you like a copy of the findings of our research?

Thank you very much for taking part.
