# Supplementary material for: Development of a novel motivational interviewing (MI) informed peer-support intervention to support mothers to breastfeed for longer
Source: BMC Pregnancy Childbirth. 2018 Apr 11;18:90. doi: 10.1186/s12884-018-1725-1 (PMC5896150; doi:10.1186/s12884-018-1725-1)
Supplement: Supplementary file 3 — Interview topic guide: health professionals and service managers. (DOCX 477 kb) [file 12884_2018_1725_MOESM3_ESM.docx]

**Motivational Interviewing based peer support for breastfeeding**

**Interview topic guide: health professionals and service managers**

**Introduction**

Thanks very much for talking to me today. We’re developing a new peer-support programme for breastfeeding and we’d really like to hear your views.

1. Could you tell me a bit about your professional role in relation to breastfeeding?
2. How are mothers supported to breastfeed in your area?

[Prompt: What about in hospital; BF support groups; peer-supporters? What support do midwives provide? What support to Health Visitors provide? Who else provides support, e.g. the voluntary sector/NHS/local authority/other?]

**Views on current peer-support provision**

1. Is peer-support for breastfeeding currently provided in your area?

[Prompts: How does peer-support work in your area? are there different peer support projects operating in the same area? How do women access these? At what point do they have contact with women, e.g. antenatal / postnatal? Do peer supporters work with mums one-to-one or in groups? Which settings do they work in (hospitals, home, community)? Who trains them? How do they link in with other breastfeeding support services?]

1. What do you think about breastfeeding peer-support?

[Do you think it’s useful? What are the best/worst things about peer-support? How could it be improved? When, where and how often should peer support be offered? Can peer support offer anything that is not covered by existing services?]

**Views on the planned MI based breastfeeding peer-support programme**

We are planning to develop a new breastfeeding peer-support service focusing on helping women breastfeed for longer. This would be an intensive one-to-one service, with peer-supporters making contact with mothers shortly before birth, within 48 hours of birth, and at the mother’s request thereafter.

The breastfeeding peer-supporters would be provided with training that includes Motivational Interviewing based approaches. This is a type of counselling that would help peer supporters to have supportive conversations with mums about breastfeeding, including talking about what is important to them and how they can achieve their goals.

1. What do you think about the breastfeeding peer-support service we’re proposing?

[Prompts: What do you think might be the advantages / disadvantages of such a service? What do you think of providing peer-supporters with training in motivational interviewing?]

1. Given the choice, would you want this type of intensive peer-support service to be offered in your area?

[Prompts: Why? What would you change? How do you think it would fit in with current breastfeeding support services in your area?]

**Implementing the breastfeeding peer-support service**

We would welcome your thoughts about how to deliver the peer support service.

1. When is the best time to provide breastfeeding peer-support?

[Prompts: What do you think about peer-supporters first meeting with mothers in the ante-natal period? What do you think about breastfeeding peer-supporters contacting mothers within 48 hours from birth? How often should peer-supporters make contact with mums? How long should they be in contact for?]

1. What barriers would there be to setting up this service in your area?

[Prompts: Are there problems we’re likely to encounter? What are the practical issues that we need to think about? Do you think there would be interest from women? Would there be interest from potential peer supporters? How might the professionals react?]

1. What could help us to with setting this programme up in your area?

[Prompts: Who are the key stakeholders? How do we engage with people?]

1. What is the best way for peer-supporters to link in with existing services in your area, such as NHS or third sector services?

[Prompts: How could we help to get the programme embedded in local services?]

1. Peer-supporters would aim to visit mums within 48 hours of the birth of their baby. What would be the best way to make sure that they’re informed when a baby has been born?
2. Because the service we’re proposing is intensive, we are considering paying peer supporters. They would be working around 15 hours a week. What do you think about this?

[Prompts: Who do you think should employ them in your area? E.g. part of NHS maternity services, the local authority, not-for-profit organisations. Dose this raise any concerns? Are there practical problems that we might encounter?]

1. How do you think peer-supporters should be recruited?

[Prompts: Are there existing services, networks, or voluntary organisations where peer supporters could potentially be recruited]

1. What should be included in initial training for peer-supporters?

[Prompts: technical/practical issues with breastfeeding; safety; reporting; communication]

1. What ongoing support should peer-supporters have when they are working in your local community (after initial training)?

[Prompts: For example, what should happen if they visit a mum who is having difficulties? Who could they go to if they have questions or need advice?]

1. Do you have any ideas about the sort of name that the peer support programme should have?

[Prompts: something breastfeeding related (bosom buddies, milk mates); something non-specific (little stars, acorns), other type of name]

1. Is there anything else we need to consider?
2. Would you like a copy of the findings?

**Thank you for taking part.**
