# Supplementary material for: Development of a novel motivational interviewing (MI) informed peer-support intervention to support mothers to breastfeed for longer
Source: BMC Pregnancy Childbirth. 2018 Apr 11;18:90. doi: 10.1186/s12884-018-1725-1 (PMC5896150; doi:10.1186/s12884-018-1725-1)
Supplement: Supplementary file 4 — Interview topic guide: focus groups with peer supporters. (DOCX 26 kb) [file 12884_2018_1725_MOESM4_ESM.docx]

**Motivational Interviewing based peer support for breastfeeding**

**Interview topic guide: focus groups with peer supporters**

Thanks very much for talking to me today. We’re developing a new breastfeeding peer-support programme that includes training peer-supporters in a counselling technique called Motivational Interviewing. We’d like to hear your views on peer-support for breastfeeding to help us design this programme.

**[**Interviewer will ask the group to introduce themselves]

1. Can you tell me a bit about your role as a breastfeeding peer-supporter?

[Prompts: When did you become a peer supporter? Are you currently supporting women to breastfeed?]

**Views about the new peer-support programme**

We’re developing a new peer-support programme to help mums who have chosen to breastfeed to do so for longer. The programme would provide mums with quite a lot of support early on. Peer-supporters would also be given training in a counselling technique called motivational interviewing, which is a way of talking. This is a technique to help peer supporters to have supportive and collaborative conversations with mums about breastfeeding, including talking about what is important to each mum and discussing how they can achieve their goals.

1. What do you think about the new peer-support programme?

[Prompts: What do you like about the idea? What do you not like about it? Can you think of any barriers to offering this kind of support? How much contact should peer-supporters have with mums? When do mums most need peer-support for breastfeeding? How do you feel about doing one-to-one sessions rather than groups?]

1. New mums who might get support from this project would be approached by their midwife at 28 weeks. If they were interested, they would be asked for permission to pass their phone number on to the peer supporter. How does this sound to you?

[Prompt: Can you think of any other ways of recruiting pregnant women? Are there problems with recruiting mums in this way? From your perspective what are the pros and cons of this approach?]

1. When do you think peer-supporters should first meet the mums they are supporting?

[Prompts: Before the baby is born? After the baby is born?]

1. At the moment, we’re thinking of asking peer-supporters to contact mums who want to use the service a few weeks before the baby is born (at about 37 weeks pregnant). What do you think about this?
2. We’re thinking of having peer-supporters visit mums within the first two days of their baby being born. How do you feel about this?

[Prompts: Would it be OK for a peer-supporter to contact mums in the first couple of days after birth? Is a home visit ok? What about in hospital? Is contact by phone or text OK?]

1. Imagine you are meeting the mum for the first time at 37 weeks pregnant, what would you like to talk to her about?

[Prompts: What would you like to know about the mum? What would you like the outcome of this conversation to be? Would you want to give the mum some information and if so what information would you like to give?]

1. We’re also thinking about asking peer-supporters to visit new mums every few days for two weeks after the baby is born. They can also keep in touch by phone or text. What do you think about this?

[Prompts: How much contact should they have? What is the best way to contact mums?]

1. After the first couple of weeks, the peer-supporter wouldn’t get in touch with mums, but mums would still be able to contact their peer-supporter if they wanted to. What are your views on this?

[Prompts: Is it OK to leave it up to the mums to make contact after the first couple of weeks? How long do mums need the more intensive support for?]

1. Imagine that your mum had decided to breastfeed her baby and she was then having second thoughts. So part of her really wanted to keep going and another part of her was just struggling with it. What would you do as a peer supporter?

[Prompt: How would you talk to her about this? Would you give her information if so what information would you give her? How would you discuss this information with the mum? What would you like the outcome of this conversation to be?]

1. Some mums may make the decision to stop breastfeeding. Imagine you have a mum who has made the decision to stop breastfeeding. What would you do as a peer supporter?

[Prompt: How would you talk to her about this? Would you give her information if so what information would you give her? What would you like the outcome of this conversation to be?]

1. Do you have any ideas about the sort of name that the peer support programme should have?

[Prompts: something breastfeeding related (bosom buddies, milk mates); something non-specific (little stars, acorns), other type of name]

1. Do you think mums would use this type of peer-support service for breastfeeding?

[Prompts: Why? Why not?]

1. What is the best way of letting mums know about the programme?
2. Would anything put mums off taking part in this programme?
3. What would encourage mums to use the programme?
4. Should partners be included in peer-support?

[Prompts: How? Should/could partners be there when peer-supporters are talking to mums? What support/information do partners need? What is the best way to get partners interested? What’s the best way to involve them? What experience have you had with partners or family members? How has that gone?]

**Recruitment and training of peer supporters**

1. We are planning to recruit 3 peer supporters in your area to work flexibly for around 15 hours a week. This might include evenings and weekends where needed. What do you think about this?

[Prompts: Do you think this would be an attractive role for peer-supporters? What would be the best way to recruit peer-supporters? Would there be any barriers to women taking up these roles?]

1. Because this would be an intensive service, we think peer supporters would need to be paid. This would probably be at a rate of about £8 per hour (or about £120 per week based on 15 hours per week). What do you think about peer supporters being paid?

[Prompt: If there was the opportunity, would you like to be paid? Do you think it would change the way you interacted with mothers if you were paid? Can you think of any issues around payment?]

1. Do you think breastfeeding peer supporters in your area would be happy to undergo extra training?

[Prompt – What would peer-supporters need to be able to undertake the training? How much training would be needed?^[[1]](#footnote-1)^ When should we hold training (weekdays/evenings/weekends)? Is it better to have shorter sessions or longer sessions (e.g. a couple of hours at a time spread over a few weeks or two full days)? What do you think about doing some training face-to-face, online, or using a portfolio? Should peer supporters to be able to bring their babies and children along to training sessions? If not, do you think we would need a crèche?]

1. What do you think is a manageable area for peer supporters to cover (in terms of location)?

*[NB: Interviewer to take local map so that peer-supporters can indicate areas]*

1. Because this new programme would be part of a research study, peer supporters would need to keep really good records of the help that they have given to women. We are thinking about asking peer-supporters to fill in a short paper-based diary (about A5 size), that would ask you to tick some boxes about the contact that you had with each mum. What do you think of this idea?

[Prompts: Is paper as best format? Would you be willing/able to complete this for every visit? Would people need for support to complete the diaries?]

1. While we were trying out the programme, we would need to look in detail at what was happening when peer-supporters visit mums. To do this, we might ask peer supporters if they could record (on a digital recorder) a couple of sessions with mums for us. How would you feel about this?

[Prompts: Are there other ways we could do this, e.g. for a researcher to come along to observe a couple of session?]

**Closing questions**

1. Do you have any other comments?
2. Would you like a copy of the findings of our research?

Thank you very much for taking part.

1. This is an important part of the economic evaluation, so please try to collect details [↑](#footnote-ref-1)
